# Construction of an alpaca immune antibody library for the selection of nanobodies against *Drosophila melanogaster* proteins

**DOI:** 10.3389/fbioe.2023.1207048

**Published:** 2023-06-09

**Authors:** Jianxiang Qiu, Jie Li, Zhen Zhang, Shirui Dong, Xiaomei Ling, Zhixin Fang, Quanshou Ling, Zhixin Huang

**Affiliations:** ^1^ Medical Research Center, Guangdong Second Provincial General Hospital, Guangzhou, Guangdong, China; ^2^ Biosafety Laboratory, Guangdong Second Provincial General Hospital, Guangzhou, Guangdong, China

**Keywords:** nanobody, *Drosophila melanogaster*, immune library, MYC, CyclinE, CG7544

## Abstract

**Introduction:** *Drosophila melanogaster* is a model organism for studying developmental biology and human neural disorders. Nanobodies are the variable domains of the heavy chains of camelid heavy-chain antibodies (VHHs) with high affinity to their antigens and have applications in basic research, similar to traditional antibodies. In addition, nanobodies acting as functionalized antibodies or protein binders have become an additional valuable approach in *Drosophila*. This study aimed to develop a VHH library against *Drosophila* proteins and confirm its availability by retrieving some *Drosophila* protein-specific nanobodies from the library.

**Methods:** An alpaca was first immunized with *Drosophila* embryo lysate and then its lymphocytes were isolated. Total RNA was extracted and cDNA was synthesized. The vhh sequences were amplified by two round PCR, which were then ligated to a phage display vector pADL-10b. The ligation products were transduced into SS320 competent cells to generate a VHH library. From this library, nanobodies against CG7544, Myc, and CyclinE was enriched and screened by phage display technology and ELISA. DNA sequences of identified nanobodies were cloned into pADL-10b-Flag-His for expression and purification in *Escherichia coli* SS320. Binding ability of purified nanobodies with corresponding antigens were determined by ELISA and surface plasmon resonance *in vitro*.

**Results:** In this study, an immune VHH library against *Drosophila* embryo proteins was generated with a capacity of 3 × 10^7^. From this library, eight nanobodies against three *Drosophila* proteins, Myc, CyclinE, and CG7544, were identified and the DNA sequences of these nanobodies were obtained. These nanobodies were successfully expressed and purified from *Escherichia coli* SS320, and were demonstrated to bind corresponding antigens with high affinity *in vitro*. Moreover, the equilibrium constant between the highest enriched nanobodies and corresponding antigens were calculated.

**Conclusion:** In summary, we report the availability of an immune VHH library and a highly efficient panning strategy for nanobodies against proteins in *Drosophila*.

## 1 Introduction

Traditional antibodies are assembled from two identical heavy-chain and two identical light-chain polypeptides (Padlan, 1994). Interestingly, the sera of camelids and sharks contain heavy chain antibodies (HCAs) in addition to traditional antibodies (Hamers-Casterman et al., 1993). HCAs consist of two identical heavy chains without light chains, and the various domains of the heavy chains of HCAs (VHHs) are able to bind antigens with high affinity (Muyldermans, 2013). The recombinant antigen-specific, single-domain VHH is known as a single-domain antibody. The molecular weight of a VHH is 12–15 kDa on average, and its size is in the nanometre range (approximately 4 × 2.5 × 3 nm) (Desmyter et al., 1996). Therefore, VHHs are also known as nanobodies.

Similar to the structure of various domains of conventional antibodies, VHHs contain four framework regions (FRs) and three complementarity-determining regions (CDRs) (Muyldermans, 2013; Mitchell and Colwell, 2018), and the CDRs of VHHs play a critical role in their stability and binding affinity (Muyldermans, 2013; Mitchell and Colwell, 2018; Liu and Yang, 2022). A VHH possesses a longer CDR3 and an additional disulfide bridge linking the CDRs (CDR1 and CDR3), which enables the formation of a new kind of loop to help bind to unique epitopes and recognize an increased variety of epitopes (Muyldermans, 2013; Mitchell and Colwell, 2018).

To date, nanobodies are considered the smallest naturally derived antigen-binding fragment and provide many advantages over conventional antibodies and their recombinant fragments Fab and scFv. The advantageous properties of nanobodies, such as their sustainable source, economical production, small size, stable and soluble behaviour in aqueous solution, and specific and high affinity, endow them with wide applications, including as research tools, as diagnostic tools, and as therapeutics. Nanobodies are easy to clone because they consist of only one domain and can be expressed with high yield and activity in bacteria or yeast (Harmsen and De Haard, 2007; Muyldermans, 2021). Nanobodies possess high solubility and refolding capacity even when exposed to extreme conditions such as very low/high pH and temperature (Ladenson et al., 2006). Moreover, the high variability in the length and sequence of VHHs and their small size allow nanobodies to efficiently enter tissues and bind epitopes that typically cannot be reached by conventional intact antibodies (Smolarek et al., 2012).

Nanobodies can be easily produced in animals of the *Camelidae* family, including camels, llamas, and alpaca, as well as some sharks (Muyldermans, 2021). Generally, a VHH library was first constructed. There are three types of VHH libraries: immune, naïve, and synthetic libraries. The retrieval of antigen-specific nanobodies from libraries involves enrichment of antigen-specific binders by phage display, bacterial two-hybrid or bacterial surface display, yeast display, or deep sequencing and subsequent screening for positive colonies from enriched phage populations by enzyme-linked immune sorbent assay (ELISA), high-throughput DNA sequencing, or mass spectrometric identification (Liu et al., 2018; Muyldermans, 2021; Liu and Yang, 2022). *In vivo* maturation of nanobodies of immune libraries and robust, fast, and versatile phage display make the combination of immune library and phage display panning by far the most common method to screen antigen-specific nanobodies, and nanobodies against >1,000 different antigens have already been isolated and characterized by this method (Wilton et al., 2018; Muyldermans, 2021). Of note, ten proteins can be mixed to immunize an animal, but even more complex mixtures (i.e., viruses, bacteria, parasites, intact mouse splenocytes, or protein extracts of cancer cells) seem to work (Thys et al., 2010; Abbady et al., 2011; Duarte et al., 2016; Odongo et al., 2016; Jovcevska et al., 2017; Muyldermans, 2021). Usually, a good immune nanobody library should have a capacity of >10^7^ individual transformants and a vhh insertion rate of at least 70% (Muyldermans, 2021). Previously, we successfully developed a nanobody selection and production platform by combining phage display technology and ELISA and identified several nanobodies specifically against green florescent protein (GFP) (Fang et al., 2020). In this study, this platform was optimized and applied for the selection of other antigen-specific nanobodies.

*D. melanogaster* is a model organism used in genetics and developmental biology, and antibodies are a preferred tool in basic research. The protein homology between *D. melanogaster* and humans is relatively low, and some antibodies against human proteins are not applicable to *Drosophila*. Moreover, nanobodies have emerged as powerful protein-binding tools to reveal protein function. Using functionalized protein binders, the protein of interest can be visualized, degraded, and delocalized *in vivo* (Harmansa et al., 2017; Kim et al., 2022; Lepeta et al., 2022). To date, nanobody tools have been used for *Drosophila* studies *in vitro* and *in vivo*. However, most of these applications use nanobodies against epitope tags or GFP to regulate corresponding fusion proteins (Caussinus and Affolter, 2016; Kim et al., 2022; Xu et al., 2022). The need for such functionalized nanobodies restricts nanobody application in developmental biology studies, and GFP inserted close to a domain of interest may affect protein function. A recent study applied a toolbox of nanobodies recognizing various domains of two *Drosophila* titin homologues (Sallimus and Projectin) to investigate protein localization and dynamics (Loreau et al., 2023). Their results found that a gigantic Sallimus isoform stretches more than 2 µm to bridge the sarcomeric I-band, while Projectin covers almost all myosin filaments in a polar orientation. *In vivo* expression of anti-Sallimus nanobodies did not fully degrade Sallimus in mature sarcomeres; however, the expression of these nanobodies caused developmental lethality (Loreau et al., 2023). This study suggests that similar nanobody tools could be applied to study other large protein complexes in *Drosophila* and mammals.

In the present study, we aimed to develop a VHH library against *Drosophila* proteins and confirm its availability by retrieving some *Drosophila* protein-specific nanobodies from the library. A VHH library was first constructed by immunizing an alpaca with *Drosophila* embryo protein lysate. Then, nanobodies against three *Drosophila* proteins (Myc, CyclinE, and CG7544) were selected individually by panning the library using phage display technology and screened by ELISA. These antibodies were expressed and purified from *E. coli* (*Escherichia coli*), and their ability to bind corresponding antigens was confirmed by ELISA and surface plasmon resonance (SPR).

## 2 Materials and methods

### 2.1 Construction and confirmation of the VHH library

Embryo lysate (0–2 h) of *D. melanogaster* was prepared and diluted to 2 mg/mL. The lysate was mixed with an equal volume of adjuvant (GERBU). An alpaca was immunized with 1 mL of mixture by 4 subcutaneous injections at fortnightly intervals performed by Shenzhen Kangti Life Technology in Shenzhen, China, as previously described (Fang et al., 2020). Isolation of lymphocytes from the immunized alpaca, RNA extraction, cDNA synthesis, and VHH library construction were conducted as described in our previous study (Fang et al., 2020). Specifically, 50 mL of peripheral blood was collected from the alpaca, and lymphocytes were isolated by Ficoll-Hypaque density gradient centrifugation. Total RNA was extracted by TRIzol Reagent, and cDNA was synthesized. Sequences encoding various domains of all heavy chains (*vh*) were first amplified by PCR. The *vhh* sequences were then amplified using the *vh* sequences as templates, which were then ligated to a phage display vector pADL-10b. Subsequently, the ligation products were transduced into SS320 competent cells to generate the VHH library.

### 2.2 Purification of *drosophila* proteins from *E. coli*

CG7544 contains 305 amino acids (aa), and the *Drosophila* Myc and CyclinE proteins consist of 717 and 709 aa, respectively. Considering the difficult expression and purification of large proteins in *E. coli*, truncated Myc and CyclinE were used in our study to screen corresponding nanobodies. Full-length CG7544 and amino acids 1–400 of Myc were PCR-amplified from *Drosophila* cDNA with the respective primer pairs 7544F: 5′-CGGGATCCATGGTAAAAACTAAGGGAAACCAAAAG-3′/7544R: 5′-CCC​AAG​CTT​TTA​TGT​TGT​CCC​CTG​CTG​TAG-3′ and mycF: 5′-CGGAATTCGCCCTTTACCGCTCTGATCCGTAT-3′/mycR: 5′-CCC​AAG​CTT​TTA​ACC​ATC​GTC​CAC​CAT​ATC​GTT​GCA​G-3′ and were cloned into pET-28a individually. Amino acids 1–361 of CyclinE were PCR-amplified from cDNA with the primers cycEF: 5′-CGGAATTCAGTGTTTGTTCTACCAGCAGCACTGAG-3′/cycER: 5′-CCC​AAG​CTT​TTA​CAA​GAG​AAT​GGC​ACG​CAT​ACG​TG-3′ and cloned into pET-28a-GFP-TEV (a plasmid constructed in our laboratory with *gfp* and tev sequences inserted into pET-28a). These expression vectors were transformed into *E. coli* BL21 (DE3), and CG7544 and Myc were purified by affinity chromatography on nickel columns in a denatured form and then refolded. GFP-TEV-CyclinE was purified in a soluble form. CyclinE was obtained by cleaving GFP-TEV-CyclinE with TEV protease and purified by affinity chromatography on a nickel column.

### 2.3 Enrichment and selection for antigen-specific VHHs

To pan for VHH binders of CG7544, Myc, and CyclinE, a phage display library was first obtained from the vhh library, and its titre was determined by preparing serial tenfold dilutions according to the methods described previously (Pardon et al., 2014). The panning process was conducted as described in our previous study (Fang et al., 2020) with some modifications. Specifically, 60 μg of CG7544, Myc, or CyclinE was incubated with 30 μL of Dynabeads His-Tag Isolation and Pulldown (Invitrogen) for 1 h at room temperature (RT). The protein Dynabeads were then washed three times with PBST (containing 0.05% Tween-20). Meanwhile, the phage display library containing 5 × 10^12^ phages in 500 μL of incubation buffer (PBST with 0.05% BSA) was pretreated by incubating with 30 μL of Dynabeads for 30 min at RT. Then, the pretreated phage display library was added to the protein Dynabeads, which were incubated with rotation at RT for 2 h. After that, the beads were washed 25 times with PBST to remove free and weakly bound phages. Finally, 500 μL of trypsin (0.25 mg/mL) was added to the beads and incubated at RT for 30 min to disassociate the binding phages. The eluted phages were neutralized with 10 μL of protease inhibitor cocktail (Roche). The first sublibrary and corresponding subphage library were obtained as previously described (Fang et al., 2020). This panning process was repeated three times to enrich phages displaying antigen-specific binders, and the number of input phages in the second and third rounds was reduced to 1 × 10^12^. Some colonies from the third sublibrary were randomly selected and sequenced.

Subsequently, ELISA was applied to select positive clones expressing antigen-specific binders from the corresponding third sub-vhh library. Specifically, a 96-well microtiter plate was coated with CG7544, Myc, or CyclinE and blocked with 3% BSA. ELISA was performed as previously described (Fang et al., 2020). Wells with an absorbance value at 405 nm two fold higher than those in negative control wells were defined as positive wells, and the corresponding colonies were defined as positive colonies.

### 2.4 Expression and purification of selected VHHs

Eight sequences encoding the potential VHHs against Myc (*a8*, *b10*), CyclinE (*n3*, *n4*, and *n6*), and CG7544 (*c3*, *c4*, and *c8*) were identified and named as indicated in the brackets. To express and purify these VHHs, a prokaryotic expression vector, pADL-10b-Flag-His, was constructed by inserting Flag- and 6 × His-encoding sequences into pADL-10b, which contains pelB sequences to secrete VHHs to the periphery. Then, the eight vhh sequences were PCR-amplified individually from corresponding phagemids using the primer pair nbF: CCG​GAA​TTC​ATG​GCA​GAT​GTG​CAG​CTG​CAG/nbR: GGC​CTC​GAG​ACC​AGA​ACC​ACC​GCT​GGA​GAC​GGT​GAC​CTG​GGT​C, digested with *Eco*RI/*Xho* I (indicated with underlines), and cloned into pADL-10b-Flag-His individually to generate pADL-10b-Flag-Nb-His. A GGSG linker (indicated with wavy lines) was inserted between the nanobody and 6× His sequences. pADL-10b-Flag-Nb-His was then transformed individually into competent SS320 for expression. VHHs were induced with IPTG at a final concentration of 0.4 mM when SS320 reached exponential phase at 37°C, and SS320 was then cultured at 30°C overnight. Crude extracts of VHHs were obtained by osmometry and then purified by affinity chromatography on a nickel column.

### 2.5 Binding ability of purified VHHs with antigens *in vitro* by ELISA

ELISA was first performed to confirm the binding ability between purified VHHs and corresponding antigens. Specifically, a 96-well microtiter plate was coated with 1 μg/well purified CG7544, Myc, or CyclinE and blocked with 3% BSA. This plate was then incubated sequentially with 1 μg of purified VHHs for 2 h at RT, anti-Flag antibody for 1 h at RT, HRP-conjugated goat anti-mouse antibody for 1 h at RT, and TMB (200 μL/well) (Thermo Scientific). TMB was incubated for no more than 30 min in the dark. The reaction was terminated with H_2_SO_4,_ and the absorbance at 450 nm was determined. As negative controls, no Flag antibody or VHHs were used. Each VHH assay was conducted in triplicate, and the results represent three independent assays.

### 2.6 Binding ability of purified VHHs with antigen *in vitro* by SPR

SPR was applied to further confirm the binding ability between purified VHHs and corresponding antigens. The SPR experiment was performed using the bScreen LB 991 Label-free Microarray System (BERTHOLD TECHNOLOGIES, Germany). The chemically modified label-free photo-cross-linker sensor chips were provided by Betterways Inc., (China). Purified CG7544, Myc, and CyclinE (in PBS) were individually immobilized onto the surface of the optical cross-linked chip. Purified C4, A8, and N4 (in PBS) were diluted separately with running buffer at concentrations of 200 nM, 400 nM, 800 nM, 1,600 nM, and 3,200 nM and were injected as flow fluid. The solvent for proteins (PBS, pH = 7.4) was crosswise tested as blank controls and background noise controls. The captured signals were monitored in real time using the PlexArray^®^ HT system (Plexera^®^ Bioscience, Beijing, China). The processing and analysis of the association and dissociation rate constants (Ka and Kd, respectively) and the equilibrium dissociation constant (KD, kd/ka) were performed using the data analysis software of the bScreen LB 991 unlabelled microarray system according to a single-site binding model (1:1 Langmuir binding) with mass transfer limitations for determination of the binding kinetics.

## 3 Results

### 3.1 Construction and confirmation of the VHH library

**FIGURE 1:**
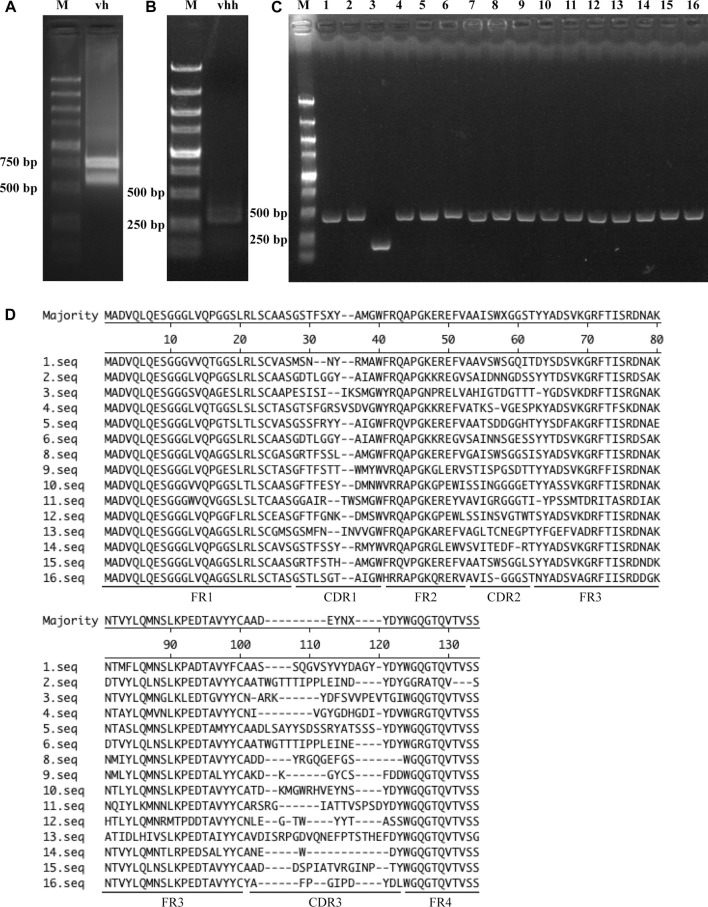
Construction and confirmation of the VHH library. **(A)** PCR amplification of the *vh* sequence. **(B)** PCR amplification of *vhh* sequences. **(C)** Insertion rate of *vhh* into pADL-10b. Sixteen colonies were randomly selected, and *vhh* sequences were amplified by PCR. **(D)** Diversity of the VHH library. Amplified *vhh* sequences were sequenced, and the FR sequences and CDR sequences of VHHs are shown.

First, an immune VHH library was constructed as described in the Materials and Methods. PCR amplification of *vh* sequences resulted in two products with lengths of nearly 800 bp and 600 bp (Figure 1A). *vhh* sequences were then amplified using the 600 bp fragment as a template, and an 400-bp product was obtained (Figure 1B). Finally, a bacterial library containing the *vhh* fragment was constructed, and its capacity was approximately 3 × 10^7^. The quality and diversity of the library were determined by PCR and DNA sequencing. As shown in Figure 1C, 15 of 16 ransom-selected colonies contained *vhh* fragments, suggesting a nearly 94% insertion rate of the library. These *vhh* fragments were further sequenced, and their amino acid sequences showed different CDRs among these colonies, suggesting a high diversity of the library (Figure 1D).

### 3.2 Screening and identification of nanobodies against specific *drosophila* proteins

**FIGURE 2:**
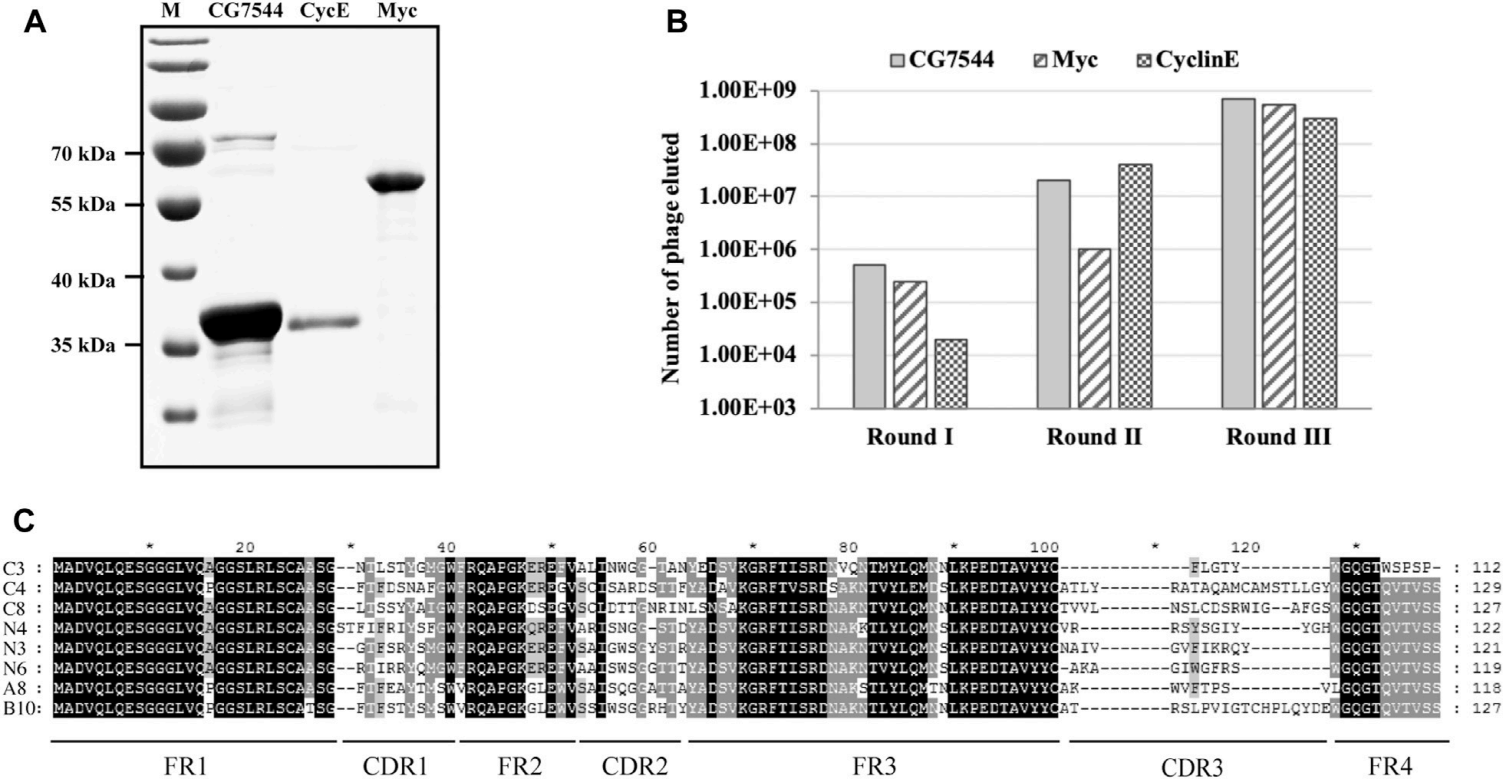
Screening and selection of antigen-specific VHHs. **(A)** Purification of Myc, CyclinE (CycE), and CG7544 from bacteria. **(B)** The output phage number in three round panning processes. **(C)** Sequence alignment of the eight antigen-specific VHHs. Amino acid sequences were aligned using ClustalX 1.83 and were edited with GeneDoc software. Black, dark grey, and light grey represent 100%, 80%, and 60% conservation, respectively. C3, C4, and C8 are nanobodies against CG7544. N3, N4, and N6 are nanobodies against CyclinE. A8 and B10 are nanobodies against Myc. FR and CDR3 sequences of the VHHs are indicated. The numbers on the right indicate the number of amino acids.

Myc encodes a transcription factor that is homologous to vertebrate Myc proto-oncogenes. It contributes to cell growth, cell competition and regenerative proliferation. CyclinE is essential for the control of the cell cycle at the G1/S (start) transition. CG7544 is the RNA N6-methyltransferase that mediates N6-methylation of adenine of U6 small nuclear RNA in *Drosophila*. They all have critical roles in *Drosophila* development. The three *Drosophila* proteins were selected as antigens to screen corresponding nanobodies from the immune library. Prokaryotic expression vectors for full-length CG7544 and truncated Myc and CyclinE were constructed, and the three proteins were purified from *E. coli* (Figure 2A). VHHs against these proteins were enriched individually for three rounds by phage display. The number of input phages was 5 × 10^12^ in the first round and 1 × 10^12^ in the second and third rounds. The number of output phages in each round is shown in Figure 2B, and those in the third round were 5.5 × 10^8^, 3 × 10^8^, and 7 × 10^8^ for Myc, CyclinE, and CG7544, respectively, showing an increasing number of output phages. These results indicated the successful enrichment of VHH binders. To estimate the efficiency of enrichment, colonies were randomly selected from the third round sublibrary of Myc, CyclinE, or CG7544, and the inserted *vhhs* were sequenced. As shown in Supplementary Figure S1, enriched sequences with the same CDR3 could be found in the VHH libraries against Myc, CyclinE, or CG7544, also suggesting the successful enrichment of VHH binders.

Subsequently, positive colonies containing VHHs against Myc, CyclinE, or CG7544 were screened from the third-round sublibrary by ELISA. Among randomly selected colonies, colonies with positive reactions were selected and sequenced. Amino acid sequences of positive colonies against Myc or CG7544 are shown in Supplementary Figure S2. Consistently, sequences of the colonies with strong positive reactions in Myc and CG7544 were the same as the most enriched sequences in Supplementary Figure S1. Randomly selected colonies (N1, N2, N3, N4, and N6 shown in Supplementary Figure S1C) from the third-round sublibrary of CyclinE were directly tested by ELISA, and N1, N3, N4, and N6 showed positive reactions. In summary, two different VHHs against Myc (A8 and B10), three different VHHs against CyclinE (N3, N4, and N6), and three different VHHs against CG7544 (C3, C4, and C8) were obtained, and their sequences are shown in Figure 2C. The DNA sequences encoding the eight VHHs were deposited in the GenBank of the National Center for Biotechnology Information, and their accession numbers are as follows: A8 [OQ822238], B10 [OQ822239], N3 [OQ822240], N4 [OQ822241], N6 [OQ822242], C3 [OQ822243], C4 [OQ822244], and C8 [OQ822245]. Moreover, the most enriched VHHs against Myc, CyclinE, and CG7544 were A8, N4, and C4, respectively. According to the results of our previous study and other studies, the most enriched VHH has a strong binding ability with its antigen (Fridy et al., 2014; Fang et al., 2020). Therefore, A8, N4, and C4 were expected to bind Myc, CyclinE, or CG7544 with high affinity.

### 3.3 Binding ability of purified VHHs with corresponding antigens by ELISA

**FIGURE 3:**
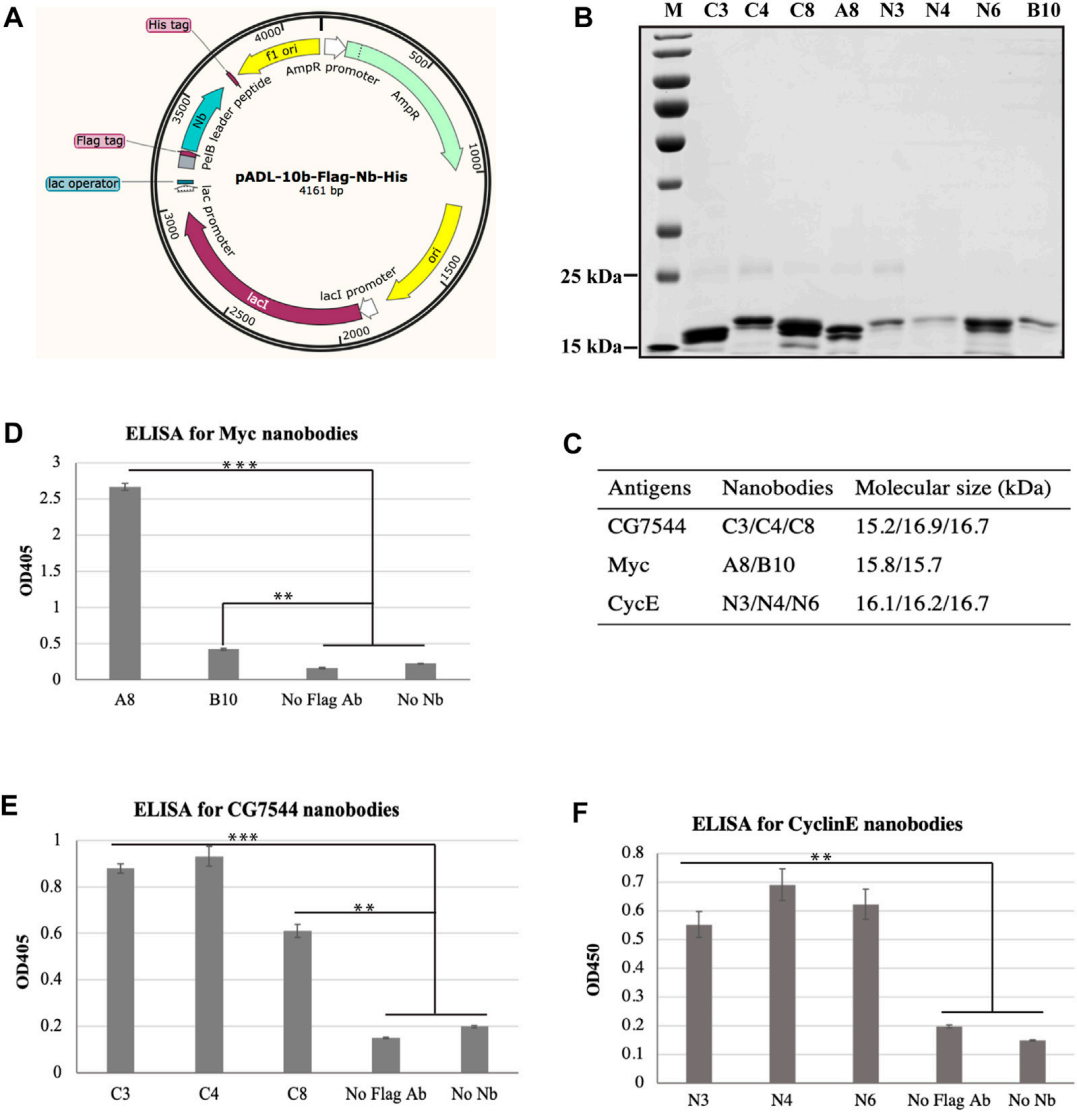
Binding analysis of purified VHHs to purified antigens by ELISA. **(A)** Schematic diagram of expression vectors. **(B)** SDS‒PAGE analysis of purified VHHs. Purified VHHs were analysed by 15% (w/v) SDS‒PAGE and stained with Coomassie Blue. The molecular weight of each VHH is shown in **(C)**. **(D–F)** Binding ability of purified VHHs with corresponding antigens by ELISA. Student’s t test was employed to determine the significant difference between samples. ** indicates *p* < 0.01 and *** indicates *p* < 0.001.

To confirm the interaction between selected nanobodies and their corresponding antigens, *a8, b10, n3, n4, n6, c3, c4,* and *c8* were first cloned into the prokaryotic expression vector pADL-10b-Flag-His (Figure 3A) individually and purified from bacteria as described in Materials and methods. The purity and molecular sizes of these nanobodies are shown in Figures 3B, C.

ELISA was then performed to determine the binding ability between purified nanobodies and corresponding purified antigens. As shown in Figures 3D–F, all nanobodies showed positive interactions with antigens. For the most enriched VHHs, the absorbance values of N3 (Figure 3E) and C4 (Figure 3F) at OD_450_ were more than 4-fold and 3-fold higher than those of the negative controls, respectively. In particular, the absorbance value at OD_450_ of A8 was more than 11-fold higher than that of the negative controls (Figure 3D), suggesting the strong binding between A8 and Myc.

### 3.4 Affinity analysis of the most enriched VHHs with corresponding antigens by SPR

**FIGURE 4:**
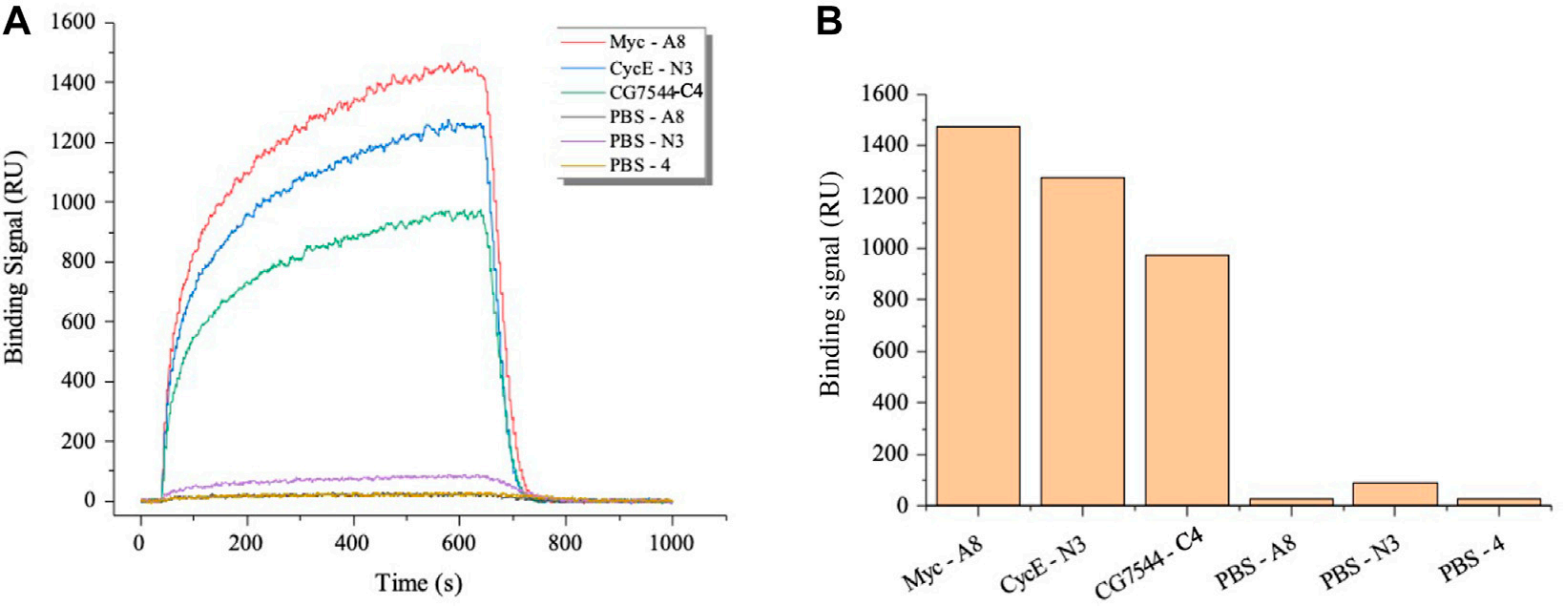
Affinity analysis of the VHHs most enriched with antigens by SPR. **(A)** Dynamic curve of the interaction between VHHs and antigens. **(B)** The maximum binding signal of VHHs with antigens. Antigens were immobilized onto the surface of the optical cross-linked chip. VHHs in PBS were injected as the flow fluid at a final concentration of 3,200 nM.

Subsequently, SPR was applied to further determine the binding affinity between nanobodies and antigens. As shown in Figure 4A, binding signals were hardly detected in the negative groups of PBS-A8, PBS-N3, and PBS-C4, while Myc-A8, CyclinE-N3, and CG7544-C4 showed strong binding signals. The maximum values of the binding signal are shown in Figure 4B. The equilibrium constants of Myc-A8, CyclinE-N3, and CG7454-C4 were 2.85E-06, 4.83E-06, and 1.05E-05, respectively, indicating strong, strong, and medium binding affinities (Table 1).

**TABLE 1:** Affinity data between purified antigens and purified nanobodies by SPR.

No.	Stationary phase	Mobile phase	Avg ka (1/Ms)	Avg kd (1/s)	Avg KD (M)	Intensity level	ABS (tr_KD)
A1	Myc	A8	1.24 E + 04	3.53 E − 02	2.85 E − 06	Strong	18.418
A2	CycE	N3	4.74 E + 02	2.29 E − 03	4.83 E − 06	Strong	17.660
A3	CG7544	C4	4.94 E + 02	5.18 E − 03	1.05 E − 05	Middle	16.540
A4	PBS	A8	1.16 E + 00	5.45 E − 01	4.72 E − 01	VW/None	1.083
A5	PBS	N4	3.71 E + 00	5.23 E − 01	1.41 E − 01	VW/None	2.825
A6	PBS	C4	1.16 E + 00	7.97 E − 01	6.88 E − 01	VW/None	0.540

Avg: average data from three independent assays of three concentration gradients.

Ka (1/Ms): association constant.

Kd (1/s): dissociation constant.

KD: equilibrium constant. KD, is the ratio of Kd/Ka. The smaller the KD, value is, the greater the binding affinity of the ligand for its target.

Intensity level: very strong affinity (10^−13^–10^−8^), strong affinity (10^−8^–10^−5^), medium or weak affinity (10^−5^–10^−2^).

ABS (tr_KD): absolute affinity coefficient. The larger the value is, the greater the binding affinity of the ligand for its target.

## 4 Discussion

Nanobodies have become a valuable new approach to investigate development in *Drosophila*. In this study, an immune library against *Drosophila* embryo proteins with a capacity of 3 × 10^7^ was successfully generated. Three highly enriched VHHs recognizing *Drosophila* Myc, CyclinE, and CG7544 were identified and named A8, N3, and C4, respectively, and their DNA sequences were obtained. The three nanobodies were demonstrated to bind corresponding antigens by ELISA and SPR.

Although no more than 30 colonies were selected for ELISA for each antigen, the most enriched and abundant VHH was identified, which suggests the high efficiency of our panning strategy. This result is consistent with our previous results, further demonstrating the high efficiency of the combination of magnetic beads, phage display, and ELISA for antigen-specific nanobody screening from a VHH library (Fang et al., 2020). According to our previous study (Fang et al., 2020), the most abundant and enriched VHH is expected to have the highest affinity for the antigen. Consistently, binding analysis of purified nanobodies with purified antigen by ELISA showed that A8 and C4 had the highest absorbance at OD_450_ nm (Figures 3D, E). For CyclinE, N3 was the most enriched during the panning process, but its absorbance at OD_450_ nm was slightly lower than that of N4 in ELISA (Figure 3F). We then assessed the binding ability of CyclinE and N4 by SPR, and the results showed weak affinity between them (Supplementary Figure S3). The lower absorbance of N3 at OD_450_ nm in ELISA may be due to the lower purity of N3 compared with N4 and N6, as shown in Figure 3B. Together, the most enriched A8, N3, and C4 have the strongest binding ability with Myc, CyclinE, and CG7544, respectively.

The KD of A8, N3, and C4 with their antigens is 10^6^, 10^6^, and 10^5^ M (Table 1), respectively, indicating strong, strong, and middle affinity (Table 1). However, it has been reported that nanobodies’ performance by SPR mostly shows a k_on_ rate of 10^5^–10^6^ M^−1^s^−1^ and a k_off_ rate of 10^3^ s^−1^, corresponding to a binding event in the nM affinity range (Muyldermans, 2021). It is possible that the immunogen in this study is a mixture of embryo proteins, and some proteins with low abundance in embryos may not stimulate strong immune reactions; therefore, nanobodies have relatively low affinity. Indeed, data in FlyBase (http://flybase.org/) (Tweedie et al., 2009) show that the level of CyclinE protein in *Drosophila* embryos is low, while no embryogenesis proteome data are available for Myc and CG7544. Nevertheless, the affinity of these nanobodies can be further improved *in vitro* by random mutagenesis and further rounds of phage display (Pleiner et al., 2018), which is the same with nanobodies retrieved from naïve and synthetic libraries. However, high-affinity nanobodies for proteins with high abundance in embryos are expected to be obtained directly from this library with the methods described in this study.

Notably, full-length Myc and CyclinE are approximately 70 kDa and therefore are truncated for better expression in bacteria, and no conserved domains were included in the truncated fragments of Myc (aa 1–400) and CyclinE (aa 1–361). Nanobodies against these fragments were successfully identified, demonstrating that this strategy is feasible and promising. A specific amino acid region or domain of a large protein is suitable for nanobody retrieval. It is easier to purify antigens in a soluble form and obtain a specific VHH. A recent study selected a subset of small domains of *Drosophila* titins (Sallimus and Projectin) as immunogens and successfully retrieved functional nanobodies from an immune library (Loreau et al., 2023). A limitation of our study is that truncated Myc and full-length CG7544 are denatured during purification, and whether all three antigens folded correctly is unclear, which may affect the diverse application of nanobodies. Further experiments in *Drosophila in vivo* or in *Drosophila* S2 cells are required to investigate the binding ability of A8, N3, and C4 to their native antigens by Western blot analysis, immunofluorescence, immunoprecipitation, or as intrabodies. Since traditional nanobodies against Myc, CyclinE, and CG7544 are lacking in our laboratory, these experiments cannot be conducted yet. Generally, denatured antigens may produce nanobodies that are not applicable in immunoprecipitation and immunofluorescence; therefore, eukaryotic expression of antigens will be preferred in our further research on retrieving function-diverse VHHs.

In conclusion, our study constructed a functional VHH library against *Drosophila* embryo proteins. Our results demonstrated that specific nanobodies can be obtained from this immune library against both truncated and full-length proteins. Naturally, purified proteins or protein fragments are supposed to be better as antigens, and corresponding functional nanobodies are easier to obtain from the VHH library. In the future, nanobodies against more *Drosophila* proteins can be retrieved from the immune library with a similar strategy.

## Data Availability

The datasets presented in this study can be found in online repositories. The names of the repository/repositories and accession number (s) can be found in the article/Supplementary Material.
