# Supplementary material for: Construction of an alpaca immune antibody library for the selection of nanobodies against Drosophila melanogaster proteins
Source: Front Bioeng Biotechnol. 2023 Jun 9;11:1207048. doi: 10.3389/fbioe.2023.1207048 (PMC10289234; doi:10.3389/fbioe.2023.1207048)

Supplementary Material

**Construction of an alpaca immune antibody library for the selection of nanobodies against Drosophila melanogaster proteins**

**Jianxiang Qiu^*^, Jie Li, Zhen Zhang, Shirui Dong, Xiaomei Ling, Zhixin Fang, Quanshou Ling^*^, Zhixin Huang^*^**

*** Correspondence:** Jianxiang Qiu: [qiujianxiang2008@126.com](mailto:qiujianxiang2008@126.com)

Quanshou ling: [657418825@qq.com](mailto:657418825@qq.com)

Zhixin Huang: [hzxd6@163.com](mailto:hzxd6@163.com)

## Supplementary Figures

**Supplementary Figure 1. Amino acid sequences of randomly selected VHHs after three round panning.** Clones were selected randomly after three round panning, and vhh fragments were PCR amplified and sequenced. Their amino acid sequences were aligned using ClustalX 1.83 and were edited with GeneDoc software. Black, dark grey, and light grey represent 100%, 80%, and 60% conservation, respectively. (A) VHH sequences against CG7544. (B) VHH sequences against Myc. (C) VHH sequences against CyclinE.


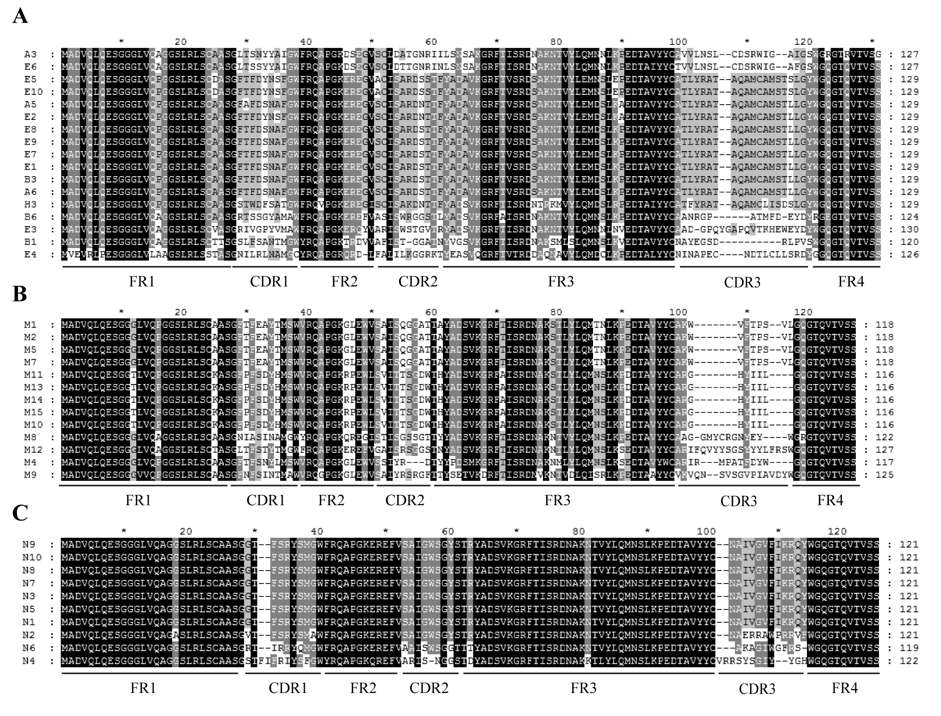


**Supplementary Figure 2. Amino acid sequences of randomly selected VHHs in ELISA.** Clones were selected randomly from the third round sub-library. VHHs were expressed by induction from this clones and used in ELISA. Clones with positive results in ELISA were sequenced. Amino acid sequences of VHHs were aligned using ClustalX 1.83 and were edited with GeneDoc software. Black, dark grey, and light grey represent 100%, 80%, and 60% conservation, respectively. (A) VHH sequences against CG7544. (B) VHH sequences against Myc.


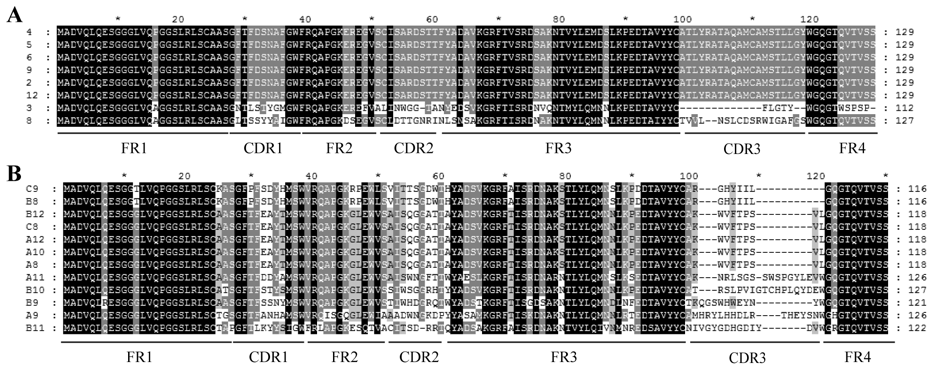


**Supplementary Figure 3. Affinity analysis between CyclinE and N4 by SPR. (A)** Dynamic curve of interaction between CyclinE and N4. (B) Dynamic curve of interaction between PBS and N4. (C) Affinity data between CyclinE and N4. CyclinE were immobilized into the surface of optical cross-linked chip. PBS was used as a negative control. N4 in PBS was injected as flow fluid at the final concentration of 200, 400, 800, 1600, and 3200 nM.


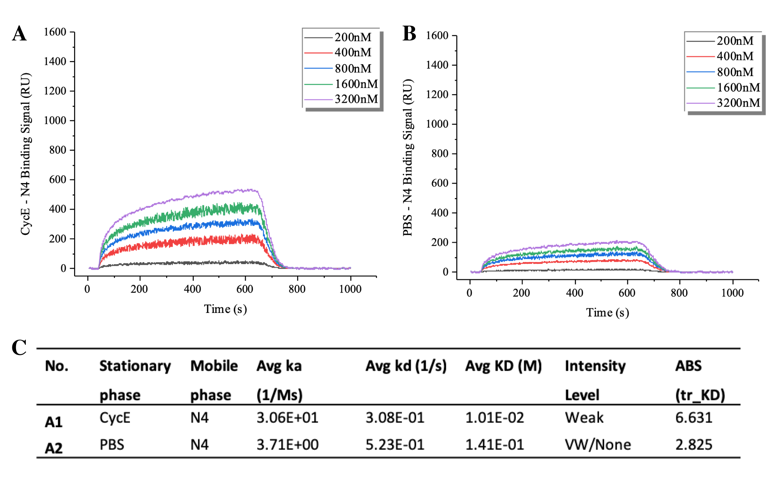

Supplement: Supplementary file 1 [file DataSheet1.docx]
